# 

**Published:** 1917-06-30

**Authors:** 


### WHEEL-CHAIRS FOR HOSPITALS.

The members of the National Deposit Friendly Society, Liverpool Division, have collected a fund for providing hospitals with wheel-chairs, in memory of fallen members of the Society. Nine chairs have now been presented; the latest was given to the Royal Southern Hospital, Liverpool, on the 4th ult.

### HOSPITAL AND INSTITUTIONAL RESULTS.

**South London Hospital for Women.**—The fifth annual report appears in the customary form, and records good progress in the new building opened by H.M. the Queen in July last. The hospital has been approved by the Local Government Board as a centre for the treatment of venereal diseases. The total income (including the Opening Day Fund, £4,584) was £8,092, and the total expenditure £4,159.

**Poplar Hospital for Accidents.**—The motto of this excellent hospital, "Never in Debt," was lived up to in 1916, when there was a surplus of £5,595, the greater part of which sum has been allocated to a suspense account for proposed buildings, alterations, and repairs at present postponed owing to the war. Only £233 was spent on renewals and repairs last year.

**Mary Yolland Home, Farnham.**—This little institution of eleven beds continues its useful work of caring for incurable children. The ordinary expenditure is about £600 a year.

**Bradford Royal Infirmary.**—The total ordinary income was £14,314, and the ordinary expenditure £17,462. Legacies were received amounting to £4,168, and donations, which for some reason were not included in the income and expenditure account, totalling £4,748 are recorded as having been received.

**Gravesend Hospital.**—The military authorities continue to make full use of the fifty beds provided for wounded soldiers, and 442 were admitted during the year. Ordinary income, £5,862; ordinary expenditure, £6,588. The report includes a sheet of photographs taken by a member of the staff on active service in Mesopotamia.

**Hospital for Sick Children, Great Ormond Street, W.C.**—The annual report has been reduced in size, and does not contain the lists of subscriptions and donations. The total income, including £2,475 legacies, was £23,978, and the total expenditure £25,032. The Working Guild of Ladies provided 1,387 garments for the patients.

**Glasgow Royal Maternity and Women's Hospital.**—The hospital at the end of the year had an overdraft of £2,380, having started 1916 with a balance in hand; this is due to a falling off in donations and legacies. Miss Schaw's large legacy of £10,000 had not been paid over when the accounts were closed.

**Wesleyan-Methodist Missionary Society.**—The 103rd report is entitled "The Story of a Great Year," and includes 212 pages, mostly descriptive of the work of the Society in different parts of the world.

### THE THEATRICAL GARDEN PARTY.

Amongst the side-shows arranged for this year's Theatrical Garden Party at the Royal Hospital Gardens, Chelsea, on July 10, is a race meeting for which a highly interesting programme has been arranged by Mr. Charles Hawtrey and Miss Vane Featherston. The "bookies'" winnings will go to swell the Theatrical Orphanage funds. The organisers of the garden party, Mr. Gerald du Maurier and Mr. Anslow Austin, are providing another attraction in the shape of a piece of the "Original Blarney Stone," which they hope may have considerable influence in helping to a solution of the Irish question.

Rates of Subscription: Twelve months, 6/6; Six months, 4/-; three months, 2/-, post free to any address in the United Kingdom. Abroad, Twelve months, 8/8; Six months, 5/-; Three months, 2/6, post free. **THE HOSPITAL "Index" to Volume LXI., October 1916 to March 1917, is now ready, price 2½d. per copy, post free.** Address The Manager, The Hospital, "The Hospital" Building, 28/29 Southampton Street, Strand, London, W.C. 2.

